# *N*-Acetyl-cysteine and Mechanisms Involved in Resolution of Chronic Wound Biofilm

**DOI:** 10.1155/2020/9589507

**Published:** 2020-01-27

**Authors:** Xin Li, Jane Kim, Jiabin Wu, Alaa' I Ahamed, Yinsheng Wang, Manuela Martins-Green

**Affiliations:** ^1^Department of Molecular, Cell and Systems Biology, University of California, Riverside, CA, USA; ^2^Department of Chemistry, University of California, Riverside, CA, USA

## Abstract

Chronic wounds are a major global health problem with the presence of biofilm significantly contributing to wound chronicity. Current treatments are ineffective in resolving biofilm and simultaneously killing the bacteria; therefore, effective biofilm-resolving drugs are needed. We have previously shown that, together with *α*-tocopherol, *N*-acetyl-cysteine (NAC) significantly improves the healing of biofilm-containing chronic wounds, in a diabetic mouse model we developed, by causing disappearance of the bacteria and breakdown of the extracellular polymeric substance (EPS). We hypothesize that NAC creates a microenvironment that affects bacterial survival and EPS integrity. To test this hypothesis, we developed an *in vitro* biofilm system using microbiome taken directly from diabetic mouse chronic wounds. For these studies, we chose mice in which chronic wound microbiome was rich in *Pseudomonas aeruginosa* (97%). We show that NAC at concentrations with pH < pKa causes bacterial cell death and breakdown of EPS. When used before biofilm is formed, NAC leads to bacterial cell death whereas treatment after the biofilm is established NAC causes biofilm dismantling accompanied by bacterial cell death. Mechanistically, we show that NAC can penetrate the bacterial membrane, increase oxidative stress, and halt protein synthesis. We also show that low pH is important for the actions of NAC and that bacterial death occurs independently of the presence of biofilm. In addition, we show that both the acetyl and carboxylic groups play key roles in NAC functions. The results presented here provide insight into the mechanisms by which NAC dismantles biofilm and how it could be used to treat chronic wounds after debridement (NAC applied at the start of culture) or without debridement (NAC applied when biofilm is already formed). This approach can be taken to develop biofilm from microbiome taken directly from human chronic wounds to test molecules that could be effective for the treatment of specific biofilm compositions.

## 1. Introduction

Chronic wounds are a major global health problem. The care for chronic wounds leads to a large financial and psychological problem to individuals and an economic burden to the society. In the United States alone, 6.5 million people suffer from chronic wounds, along with the cost of $25 billion per year spent in wound care [1]. Chronic wounds are commonly found in situations involving obesity, vascular diseases, and aging [2]. In these wounds, reepithelialization does not occur in a timely and orderly manner; the wounds stall in the inflammatory phase with excessive levels of reactive oxygen species (ROS), cytokines, and proteases; increased cell death, persistent infections, and lack of microvessel development occur [3–6]. Moreover, defective mesenchymal stem cells [7–9], degradation of growth factors [10], diminished capacity of fibroblast proliferation and migration [11], and downregulation of wound-associated keratin and its heteropolymers [12] result in impairment of healing. These conditions promote pathogen colonization and strong biofilm formation in the wound bed, which then further delay the healing [13, 14].

Basic steps in wound care include debridement to remove biofilm, topical and/or systematic antibiotic treatment, and application of various wound dressings [15]. However, wound biofilm is persistent and difficult to eradicate. Even though surgical debridement removes existing biofilm, fresh biofilm reappears within 2-3 days, given that bacteria persist within the margin of the wound tissue even after extensive debridement [16]. Antibiofilm drugs that can either facilitate the dispersion of preformed biofilm or inhibit the formation of new biofilm are an unmet need. There are numerous potential antibiofilm treatments under investigation. For example, when topically applied, acetic acid, boric acid, ascorbic acid, alginic acid, and hyaluronic acid improve the wound healing process and decrease infections [17, 18]. More recently, novel potential approaches, such as quorum sensing inhibitors that have unique structures compete with signal molecules and interfere with biofilm formation. Also, peptidomimetics that disturb bacterial membrane structure and nanoparticles that transport drugs into bacterial cells hold great promise in dismantling existing biofilms or preventing biofilm formation [19–23]. However, the field is in need of new treatments that not only are effective against bacteria but also promote wound healing and are without significant side effects.

We have recently shown that NAC applied to chronic wounds in a diabetic mouse model we have developed improved healing dramatically within 20 days of initiation of treatment [24].

However, the mechanisms by which NAC affects the extracellular polymeric substances (EPS) and interferes with bacterial function that lead to biofilm dismantling are not well understood. To study the mechanisms of action of NAC on biofilm initiation, development, and integrity, we established an *in vitro* system that leads to the development of biofilm by culturing microbiomes collected from chronic wounds. This system does not use laboratory-cultured bacteria; the bacteria are taken from biofilm collected directly from chronic wounds that we create in our db/db^−/−^ mouse model. In the studies presented here, we chose to study biofilms established *in vitro* from microbiome taken from a chronic wound that contained primarily *Pseudomonas aeruginosa.* We hypothesize that NAC creates an environment that disrupts wound microbiome biofilm by interfering with both bacterial function and survival and by disrupting biofilm EPS integrity. Our results show that NAC treatment of the biofilm *in vitro* resulted in bacterial cell death and disruption of the EPS in the biofilm. These effects were primarily accomplished by an increase in oxidative stress in the bacteria, inhibition of protein synthesis, and a decrease in the amount of DNA and proteins in the EPS. For these effects, the pH of NAC and specific functional groups of this molecule are critical. These results provide insight into the mechanisms involved in the ability of NAC to prevent chronic wound biofilm initiation and development as well as in disrupting existing biofilm. Moreover, this approach can be taken to develop biofilm from microbiome taken directly from human chronic wounds to test molecules that could be effective for the treatment of specific biofilm composition.

## 2. Methods and Materials

### 2.1. Ethics Statement

All animal experimental protocols were approved by the University of California Riverside (UCR), Institutional Animal Care and Use Committee (UCR-IACUC). All animal experiments took place in PI's (M. Martins-Green) lab at UCR. Mice were euthanized by carbon dioxide (CO_2_) inhalation, which is the most common method of euthanasia used by NIH. The amount and length of CO_2_ exposure were approved by UCR-IACUC. The animals are cared every day, 7 days a week. UCR is fully accredited by AAALAC, complying with the Guide for the Care and Use of Laboratory Animals, The Public Health Service Policy, and the Humane Care and Use of Laboratory Animals. The UCR campus veterinarian was available to assist us with any problems with the animals.

### 2.2. Collection and Storage of Wound Microbiome

Chronic wounds were made in db/db^−/−^ mice as previously described [24]. Six to seven-month-old db/db^−/−^ mice were treated intraperitoneally with a catalase inhibitor, 3-amino-1,2,4-triazole, at 1 g/kg body weight before wounding. Immediately after wounding, they were treated once topically with the glutathione peroxidase inhibitor, mercaptosuccinic acid, at 150 mg/kg body weight. Using our procedure, the wounds are fully chronic in 20 days and contain abundant biofilm. All bacteria forming the biofilm are from the mouse natural skin with no additional manipulation or introduction of laboratory-carried strains; therefore, the biofilm forms naturally from the microbiome in the skin. For these studies, the biofilms were collected using the Levine method with sterile cotton swabs and stored for bacteria identification both dry at -80°C and in Luria-Bertani (LB) media (10 g tryptone, 5 g yeast extract, and 10 g NaCl and Milli-Q water to 1 L; Teknova) supplemented with 20% glycerol. Swabs in LB media with 20% glycerol were then cultured in fresh LB media overnight at 37°C (150 rpm) and aliquots stored at -80°C.

### 2.3. Bacteria Identification in the Wound Biofilm

Bacterial species in swabs and their abundance were analyzed by extracting bacterial DNA with the PowerSoil DNA Isolation Kit (Cat. no.12888-50) as described by the manufacturer. For library construction, the ITS region was amplified using bacterial ITS rRNA primers (ITS-1507F GGTGAAGTCGTAACAAGGTA, ITS-23SR GGTTBCCCCATTCRG) with specific barcode sequences for each sample. Then, PCR products were purified with QIAGEN MinElute 96 UF PCR Purification Kit (Cat. no. 28053). The amounts of DNAs were normalized for each sample and sent to the Illumina MiSeq sequencer. The sequences were identified and organized into an OTU table generated via Qiime, an open-source bioinformatics pipeline, analyzing raw microbiome DNA sequencing data [25].

### 2.4. *In Vitro* Chronic Wound Bacterial Biofilm Model

Although the mice are genetically identical, we found that, when the wounds are fully chronic, each mouse ends up with its own biofilm-forming bacteria mostly composed by 1 or 2 such bacterial species. The most predominant species are *Pseudomonas*, *Staphylococcus*, *Acinetobacter*, and *Enterobacter*. For these studies, we chose a biofilm from a wound that contained 97% *P. aeruginosa.* This biofilm was grown in LB at 37°C with shaking (150 rpm) overnight and then inoculated in media of 96-well flat microtiter plates (Cat. no. CLS3595, Corning Incorporated, USA) or 35 mm petri dishes (Cat. no. 353001, Corning Falcon, USA) to form a biofilm. An opaque film was formed on the air-liquid interface and on solid-liquid interface attached to the sides and bottom of the well. The air-liquid biofilm can be seen 12 h after inoculation at 37°C and is fully developed by 24 h of incubation.

### 2.5. Bacteria Identification in the *In Vitro* Wound Biofilm

To determine whether this biofilm *in vitro* has the same composition as the biofilm in the original wound biofilm, we used the API 20E identification kit (REF 20 100, bioMérieux, USA) which is a standardized identification system for Enterobacteriaceae and other nonfastidious, Gram-negative rods; it uses 21 miniaturized biochemical tests and a database. The kit has a table of bacteria that can be identified, which includes *Enterobacter, Pseudomonas*, and many gram-negative bacteria. 24 h biofilm inoculum, including air-liquid interface and solid-liquid interface biofilm, was collected and sonicated for 10 s with 15% amplitude. Bacterial cells were collected by centrifugation, plated on tryptic soy agar plates (Cat. no. 236950, BD Difco, Sparks, USA) containing 5.0% (*v*/*v*) defibrinated sheep blood (Cat. no. R54008, Thermo Fisher Scientific, USA) with serial dilutions, and cultured for 12-24 h at 37°C. Three morphologically different colonies were observed; we picked five colonies of each type from the plates and analyzed them with API 20E strips consisting of 20 microtubes with dehydrated substrates. According to the manufacturer's instructions, a single colony was removed and resuspended in 0.85% NaCl solution. The suspension was distributed into the tubes of the strip and cultured at 37°C for 18-24 h, within an incubation box with 5 ml demineralized water to create a humid atmosphere. During the incubation, metabolism of the bacteria produces color changes spontaneously that can be revealed by the addition of reagents. The tests were separated into groups of 3, and a value of 1, 2, or 4 was assigned to each. After the incubation, the values were added together corresponding to positive reactions within each group. A 7-digit profile number was obtained for the total 20 tests. The bacteria species identification can be determinate by referring to the apiweb™. “API 20 E is a standardized identification system for Enterobacteriaceae and other nonfastidious, Gram-negative rods which uses 21 miniaturized biochemical tests and a database.”

### 2.6. NAC and Its Derivatives Used for This Study

*N*-Acetyl-cysteine (NAC, Cat. no. A7250-100G, Sigma-Aldrich), *N*-acetyl cysteine amide (NACA, Cat. no. A0737-5MG, Sigma-Aldrich), *N*-acetyl serine (NAS, Cat. no. A2638-1G, Sigma-Aldrich), cysteine (Cat. no. 168149-2.5G, Sigma-Aldrich), and glutathione (PHR1359-500MG, Sigma-Aldrich) were used. These molecules were dissolved in LB broth, and filter-sterilized. For the liquid culture in 96-well microtiter plates and 35 mm petri dishes, chemical solution with a final concentration (1x) was added at specific time points against the side of the well by slowly pipetting without disturbing the biofilm. Plates were continually cultured at 37°C. The condition of the surface biofilm was recorded digitally.

For chronic wound microbiome biofilm on solid surfaces, 5 *μ*l diluted inoculum (ratio 1 : 100) was added on agar for 7 h. NAC solutions (5 *μ*l) were added directly either on the top of the colonies or on the bacterial colony patches (200 *μ*l) after the culture rings were placed. The effect of NAC under these conditions was observed in 24 h or 48 h.

### 2.7. Biofilm Quantification Assays

A modified microtiter plate test for the quantification of biofilm formation was used based on a previous assay [26]. Briefly, 0.1% of a crystal violet (CV; Cat. no. C0775-25G, Sigma-Aldrich, USA) staining solution was prepared in Milli-Q water and filtered through a 0.45 *μ*m filter (Cat. no. SLHA033SS, MF-Millipore). The medium and unbounded cells were removed by pipetting. The remaining biofilm was staining in 225 *μ*l per well of 0.1% CV solution for 5 min. Staining solution was removed by pipetting. 300 *μ*l of 95% of ethanol was added to remove the staining from the biofilm. After 45 min to 1 h of incubation at room temperature to fully distain the biofilm, the absorbance was measured at 590 nm using a microtiter plate reader (Epoch Microplate Spectrophotometer, BioTek). If the reading signals were overload, distaining solutions were diluted accordingly and the appropriate multiplier was applied.

### 2.8. Biofilm Protein and DNA Analysis

24 h-biofilm cultured in 96-well microtiter plates is treated with various concentrations of NAC for 24 h. The same amount of culture for each NAC treatment was collected and briefly sonicated for 10 s with 15% amplitude. This step is to loosen the EPS structure. Samples are saved as a *whole biofilm fraction*. Cultures were centrifuged at 4°C (15,000 rpm) for 10 min. The supernatant was transferred to a new microcentrifuge tube and saved as the soluble *EPS fraction*. 30 *μ*l of whole biofilm fractions and EPS fractions was analyzed on 12% SDS-PAGE gels for protein evaluation; 10 *μ*l of whole biofilm fractions was analyzed on 1% agarose gels for DNA evaluation.

### 2.9. NAC Intracellular Assay

24 h-biofilm was treated with NAC for 24 h. Biofilm culture was collected, briefly sonicated for 10s with 15% amplitude, and centrifuged at 4°C (15,000 rpm) for 5 min. The supernatant was discarded, and the cell pellets were washed with 1 ml 1x PBS and resuspended in 0.5 ml 1x PBS. Cells were lysed by sonication and centrifuged at 15,000 rpm for 10 min. The supernatant was filtered (pore size: 0.22 *μ*m) and analyzed by HPLC. The liquid chromatography method was performed on an Agilent 1100 system with the Phenomenex Luna C_18_ column (Torrance, USA) (150 × 4.6 mm; 5 *μ*m, 100 Å). The mobile phase consisted of 0.05 M KH_2_PO_4_ (solution A) and acetonitrile (95 : 5) with 0.095% of phosphoric acid (solution B), with 3% B phase isocratic elution at a flow rate of 1.3 ml/min. The sample volume injected was 100 *μ*l, and NAC was detected at 214 nm. All analyses were performed at room temperature.

### 2.10. Measurement of Biofilm Bacteria NAD^+^/NADH Ratio

NAD^+^/NADH-Glo Assay Kit (Promega Co., Cat. no. G9072) was used to quantify NAD^+^ and NADH individually. The 24 h-biofilm was treated with NAC for 24 h. 600 *μ*l biofilm inoculum including air-liquid interface and solid-liquid interface biofilm was collected, washed twice with 1 ml 1x PBS, and resuspended with 600 *μ*l 1x PBS. To lyse the cell membrane, 200 *μ*l of bacterial cells was mixed with 200 *μ*l base solution (100 mM sodium carbonate, 20 mM sodium bicarbonate, 0.05% TritonX-100) supplemented with 1% DTAB and vortexed for 15 s. To detect the amount of NADH, 200 *μ*l of the 400 *μ*l mixture was directly incubated at 60°C for 15 min (A). The other 200 *μ*l of the mixture was mixed with 100 *μ*l 0.4 N HCl first and then incubated at 60°C for 15 min to measure the amount of NAD^+^ (B). Both A and B were equilibrated at room temperature for 10 min. Then, 200 *μ*l HCl/Tris solution (prepared by mixing the same volume of 0.4 N HCl mixed with equal volume of 0.5 M Tris base) was added to A; 100 *μ*l 0.5 M Tris base was added to B. For each NAC treatment, technical duplicates were applied for both NAD^+^ and NADH detection. 100 *μ*l of either solution A or solution B was added to a 96-well microtiter plate, and 100 *μ*l NAD^+^/NADH-Glo detection reagent was added to each well. After 45-50 min incubation at room temperature, luminescence was detected by a Promega Multi Reader.

### 2.11. Protein Synthesis Determination Using 35S Labeling

24 h-biofilm bacteria were collected by centrifugation at 15,000g for 5 min. The pellets were resuspended in LB containing either 5 mg/ml or 20 mg/ml NAC, together with the exposure of 50 *μ*Ci EXPRE^35^S^35^S Protein Labeling Mix (PerkinElmer, Cat. no. NEG072002MC). The mixtures were incubated at 37°C (225 rpm) for 4 h and then at 99°C for 5 min to kill the bacteria and stop cellular protein synthesis. Cell pellets were centrifuged at 15,000g for 5 min, washed with 1 ml 1x PBS, resuspended in 200 *μ*l 1x PBS, and analyzed on 10% SDS-PAGE gel. After staining and distaining, the SDS-PAGE gel was wrapped in a cling film and exposed to an X-ray film at 4°C for 72 h.

### 2.12. Fluorescence Staining and Confocal Laser Scanning Microscopy Analysis of the Biofilm

24 h-biofilm was prepared in the air-liquid interface of 35 mm diameter glass bottom dishes (WillCo Well, Cat. no. GWST-3522) and treated with NAC solutions for 24 h. The staining process was optimized based on a previous study [27]. Culture media were gently removed, and the biofilm was rinsed with 1 ml 1x PBS twice. All the staining processes were done at room temperature. Calcofluor White Stain solution in 1x PBS (Sigma-Aldrich, Cat. no. 18909-100ML-F) to stain polysaccharide in the biofilm was added to each plate and incubated for 20 min. The staining solution was removed, and the biofilm was rinsed with 1 ml 1x PBS. Then, the biofilm was stained with FilmTracer SYPTO Ruby Biofilm Matrix Staining solution for 30 min to label proteins in the biofilm matrix (Thermo Fisher Scientific, Cat. no. F10318). The staining solution was removed, and the biofilm was rinsed with 1 ml 1x PBS. Lastly, the biofilm was stained with SYTO9 Green Fluorescent Nucleic Acid Stain solution for 30 min to stain biofilm DNA (2.5 *μ*l of SYTO 9 Green Fluorescent Nucleic Acid Stain in 397.5 *μ*l 1x PBS). The staining solution was removed, and the biofilm was rinsed with 1 ml 1x PBS. The structure of the biofilm was observed using a confocal laser scanning with the Zeiss880 Inverted UV Spectral Airyscan Microscope. Channel mode visualization was done using the 20x objective. During the image collection, confocal tiles were obtained using channel mode with 3-track: Ex/Em 355 nm/433 nm for Calcofluor White Stain, Ex/Em 280 nm, 450 nm/610 nm for FilmTracer SYPTO Ruby, and Ex/Em 488 nm/523 nm for SYTO9 Green.

### 2.13. Statistical Analysis

For the statistical analysis of data, we used GraphPad Prism 6 software. The analysis of variance (ANOVA) was used to test the significance of group differences between two or more groups. In the experiments with only two groups, statistical analysis was conducted using Student's *t*-test.

## 3. Results

In this study, we investigated the effects of NAC on biofilm developed from the microbiome taken from chronic wounds. We have previously developed a db/db^−/−^ mouse model for chronic wounds in which the wounds become chronic, presenting all the abnormalities found in human chronic wounds, including the presence of a biofilm with bacteria that are found in chronic wounds in humans [24]. In our model, bacteria are never introduced in the wounds from external sources; the biofilm develops from the bacteria in the skin in the presence of high levels of oxidative stress [24]. In this study, to make the biofilm *in vitro*, we chose to use wound biofilm that contains primarily *P. aeruginosa* because *P. aeruginosa* colonizes about 52.2% of the patients with chronic ulcers [28, 29]. It produces strong biofilm and quickly acquires antibiotic resistance leading the wounds to become chronic [30]. Although NAC has been used to help treat mucus in cystic fibrosis, the mechanisms by which NAC dissolves biofilm are not well known and NAC has not been used to treat human chronic wounds containing *P. aeruginosa*. Below, we present data describing how we developed the *in vitro* biofilm system and how we tested the effects of NAC on biofilm initiation and development and on resolution/dismantling of established biofilm. Mechanistically, in order to kill the bacteria, NAC needs to enter the bacteria itself and, for that, low pH is critical. When inside the bacteria, NAC creates high levels of oxidative stress and inhibits protein synthesis. In addition, we also tested the importance of the various functional groups of NAC in these processes something that has never been shown previously.

### 3.1. Development of an *In Vitro* System to Study the Effects of NAC on Biofilm

**Figure 1:**
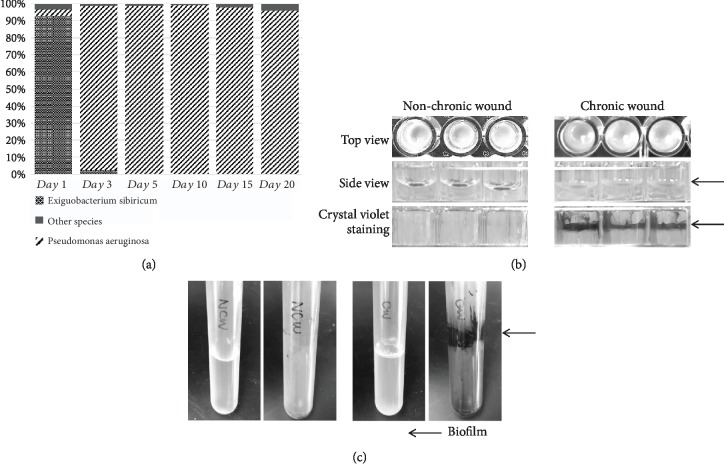
Chronic wound microbiome rich in *P. aeruginosa* formed strong biofilm *in vitro*. Microbiome rich in *P. aeruginosa* was collected from chronic wounds and cultured *in vitro* overnight. Similar cultures were performed with microbiome collected from nonchronic wound. (a) Bacterial species sequencing data using the 16S ITS region as the probe. In this particular animal, the wound became colonized with *P. aeruginosa* as early as day 3 postwounding and induction of chronicity. (b) Microbiome cultures in 96-well microtiter plates. Top view, side view, and side view with crystal violet staining. Microbiome from chronic wounds formed thick opaque biofilm on the air-liquid interface that can be stained with crystal violet (long arrow). Microbiome from nonchronic wounds did not form biofilm. Technical replicas of three wells of each condition. (c) Likewise, when cultured in tubes, microbiome from nonchronic wounds did not stain with crystal violet (left 2 tubes) whereas that from chronic wounds was stained with crystal violet (right 2 tubes).

Microbiome was directly collected from chronic wounds in our db/db^−/−^ mouse model of chronic wounds, using the methods described in the Methods and Materials section [24]. For these studies, we chose a swab taken from a fully chronic wound (20 days after wounding) with a microbiome that contained ~97% of *P. aeruginosa* (Figure 1(a)). The microbiome composition in the fully chronic wounds varies from animal to animal much likely in human subjects. To recreate a biofilm, this microbiome was cultured in 96-well microtiter plates, 35 mm petri dishes, or culture tubes. The microbiome from chronic wounds formed strong biofilm overnight in both microtiter plates and culture tubes. Microbiome from nonchronic wounds did not develop biofilm *in vitro* under the same conditions. The latter microbiome, however, contained only ~18% *P. aeruginosa* among many other bacteria species normally present in the skin (unpublished results). To make the biofilm more easily visible, both cultures were stained with crystal violet, a stain used to reveal biofilm. In both cases, the biofilm was only visible in the cultures from the microbiome taken from chronic wounds (Figures 1(b) and 1(c)).

### 3.2. Effects of NAC on Biofilm Development and Bacterial Growth

**Figure 2:**
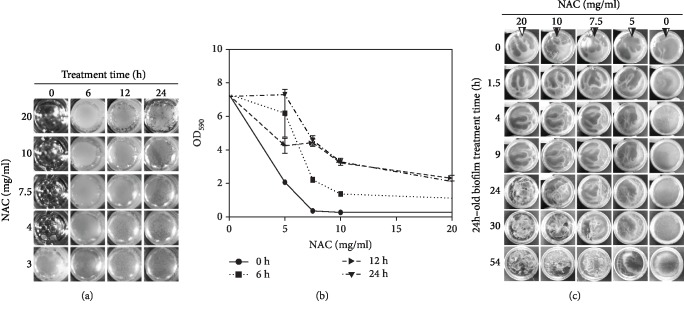
The effect of NAC on biofilm development and dismantling. (a) Microbiome taken from the chronic wounds and grown in 96-well microtiter plates. Concentrations of NAC ranging from 3 to 20 mg/ml were applied to the cultures at the times indicated (0, 6, 12, and 24 h). The pictures shown were taken at 48 h after initiation of the culture. When ≥4 mg/ml NAC were applied to the culture at 0 h, the biofilm never developed. When ≥10 mg/ml of NAC was applied at 6, 12, and 24 h after culture initiation, biofilm formation was visibly altered or became fragile and disrupted. (b) At 48 h, biofilm was stained with crystal violet (CV) and the absorbance at 590 nm was measured to quantify biofilm biomass. At 0 h, 5 mg/ml application, 5 mg/ml NAC significantly reduced the appearance of biofilm, and when applied at 6 and 12 h, it was able to decrease biofilm production but not when applied at 24 h. ≥10 mg/ml NAC or more significantly diminish biofilm formation. Three technical repeats with standard deviation as error bars for quantitative data. (c) The dose-dependent effect of NAC on 24 h old biofilm cultured in 35 mm petri dishes was recorded over time. 20 mg/ml of NAC dismantled biofilm by 24 h; 10 and 7.5 mg/ml of NAC were able to fully disrupt biofilm at 30 and 54 h, respectively. 5 mg/ml NAC did not cause changes in existing biofilm.

To determine the concentrations of NAC that inhibit biofilm development or that dismantle established biofilm created with the microbiome taken from chronic wounds, different concentrations of NAC ranging from 3 to 20 mg/ml were applied at 0, 6, 12, and 24 h after the cultures were initiated. Representative pictures of the cultures were assembled for comparison (Figure 2(a)). With the chronic wound microbiome, when ≥4 mg/ml NAC were added to the cultures at time 0 h and observed at 48 h later, there was no bacterial growth or biofilm formation. When ≥10 mg/ml NAC were added at 6, 12, or 24 h when biofilm was already visibly formed and observed at 48 h, the biofilm appeared as a thin layer and became fragile and fragmented. We also stained the biofilm with crystal violet; the amount of biofilm present was quantified after distaining with 95% ethanol to release the staining from the biofilm so that it could be quantified by reading absorbance at 590 nm (Figure 2(b)). ≥7.5 mg/ml NAC concentrations used at 0-24 h reduced the biofilm by 37-95% whereas 5 mg/ml NAC was not able to interfere with existing biofilm.

To study the efficiency of NAC in disrupting biofilm, we used 35 mm culture dishes, cultured the chronic wound microbiome for 24 h to form mature biofilm (Figure 2(c)), and then applied NAC at the various concentrations as used in the previous experiment, to one side of the plate (arrowhead). The condition of the biofilms was recorded up to 54 h. Compared to the control group, 20 mg/ml NAC significantly dismantled the chronic wound biofilm within 24 h treatment; 10 mg/ml and 7.5 mg/ml NAC were less effective at 24 h but had similar effects at 30 h and 54 h, respectively. 5 mg/ml NAC were unable to visibly disrupt the biofilm structure.

**Figure 3:**
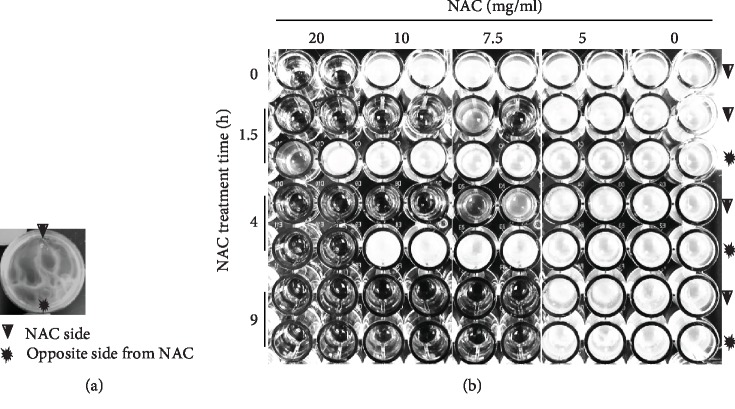
The effect of NAC on the growth of bacteria contained in biofilm. We used the culture shown in Figure 2(c) to perform this experiment. (a) NAC was applied to the culture from one side of the culture dish marked with an arrowhead. The other side of the plate where NAC was not applied is marked by a star. (b) Efficiency of NAC in penetrating the biofilm and interfering with bacterial cell growth. After 0, 1.5 h, 4 h, and 9 h NAC treatments, cells from the side where NAC was applied (arrowhead) and away from the site of application (star) were subcultured in fresh LB in a microtiter plate. 20 mg/ml NAC were able to penetrate the culture and interfere with cell growth within 1.5 h, and no cell growth was detected after 4 h treatment. 10 and 7.5 mg/ml NAC were able to penetrate the culture and completely inhibit further cell growth after 9 h post application. 5 mg/ml NAC could not inhibit cell growth. Technical duplicates were used for cell viabilities after being treated with different concentrations of NAC.

To test whether the effects of NAC on biofilm disruption were due to NAC interfering with bacteria cell growth, we grew the bacteria from the cultures shown in Figure 2(c). Due to the protection conferred by the biofilm EPS, NAC could not penetrate the entire culture immediately. For each culture, we grew the bacteria from both sides of the petri dishes: from the side where NAC was applied (arrowhead) and from the opposite side where NAC was not applied (star) (Figure 3(a)). At the time of application (0 h), NAC 20 mg/ml affected the bacteria in a way that they could no longer grow whereas treatment with ≤10 mg/ml was unable to affect bacterial growth. At 1.5 h posttreatment of the biofilm, cells taken from the treated side (arrowhead) did not grow with treatment of 7.5-20 mg/ml whereas from the nontreated side (star) they did. At 4 h of treatment, the same results were observed for the treated side as at 1.5 h treatment. However, for the nontreated side, the 20 mg/ml NAC was now affecting the growth of the bacteria. By 9 h of treatment, the 7.5-20 mg/ml NAC had spread throughout the entire petri dish leading to no growth of the bacteria. 5 mg/ml NAC were unable to stop bacterial growth on either side of the petri dish (Figure 3(b)). These results suggest that over time treatment of the biofilm with 7.5-20 mg/ml NAC stops bacterial growth and eventually dismantles the biofilm. We also investigated the effect of NAC on biofilm-free bacterial growth and found that NAC is also effective in the absence of biofilm (Supplementary material and Figures [Supplementary-material supplementary-material-1]).

### 3.3. NAC-Induced pH Changes Are Important for Its Antibiofilm Activity

**Figure 4:**
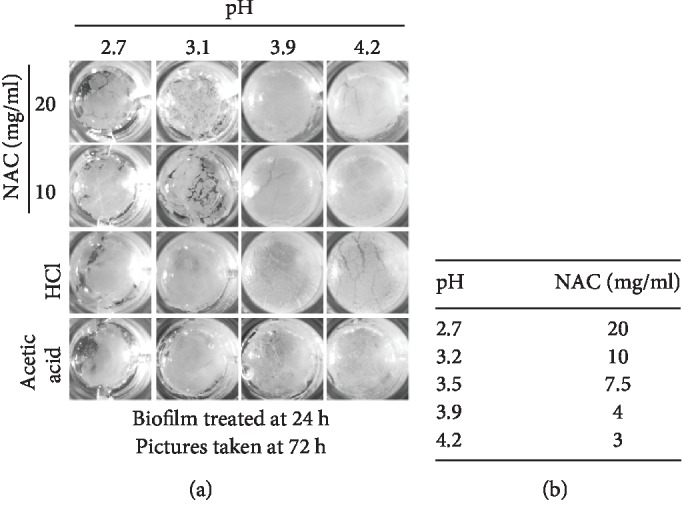
The effects of pH on the ability of NAC to dismantle the biofilm. (a) 24 h old biofilm was treated with either 20 or 10 mg/ml of NAC or HCL or acetic acid at different adjusted pH to 2.7, 3.1, 3.9, and 4.2, corresponding to the pH range of 20-3 mg/ml of NAC. When the pH of the NAC solution for both concentrations is below the pKa (3.24), biofilm was disturbed. However, when pH > pKa, the structure of the biofilm remained intact. Biofilm treated with HCL and acetic acid remained intact at ≥pH 3.1. When pH further decreased to pH 2.7, biofilm treated with both acids became fragile. (b) shows the pH of the NAC solution at the specific concentration of NAC. Three biological replicates were performed.

NAC is a weak organic acid with a pKa of 3.24. The pH of LB broth containing concentrations of 3-20 mg/ml of NAC varies from 4.2 to 2.7, respectively (Figures 4(a) and 4(b)). To identify whether the effect of NAC in dismantling biofilm is associated with pH, we replaced the LB in 24 h cultures, which contains fully developed biofilm, with LB-NAC solutions at different pH. For 20 mg/ml NAC, the pH was adjusted from 2.7 to 3.1, 3.9, and 4.2 with sodium hydroxide, and for 10 mg/ml NAC the pH was adjusted from 3.1 to 2.7 with HCl and to 3.9 and 4.2 with sodium hydroxide. We also used a strong acid, HCl, and a weak acid, acetic acid, with adjusted pH as we did with NAC. NAC 20 and 10 mg/ml at pH 2.7 and 3.1, which are below NAC pKa, disrupted the surface biofilm whereas at pH 3.9 and 4.2, which are above the pKa, did not. Both acetic acid and HCl at pH 2.7 disrupted the surface biofilm albeit to a lesser extent than NAC at the same pH, and they did not disrupt the biofilm at higher pH (Figure 4(a)). These results suggest that pH is not the only factor involved in biofilm disruption by NAC and that NAC has additional properties that affect the integrity of the biofilm either by affecting the bacteria or by disrupting the EPS, or both, leading to dismantling of the biofilm.

### 3.4. At pH below the pKa, NAC Penetrates the Bacterial Cell Membrane, Increases Oxidative Stress, and Inhibits Protein Synthesis

**Figure 5:**
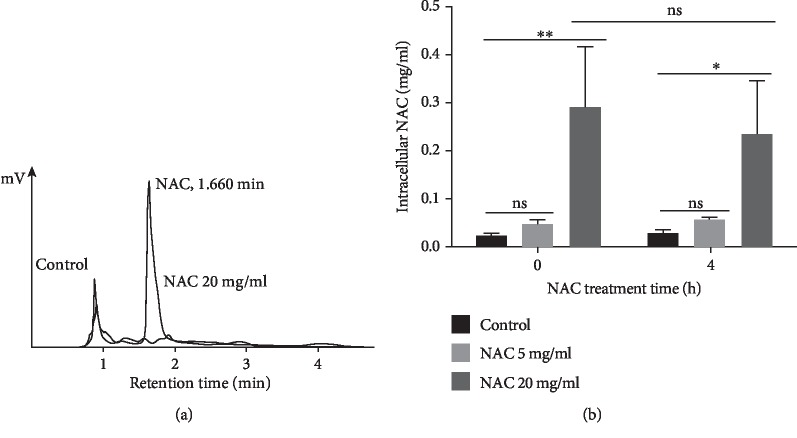
Detection of intracellular NAC using HPLC. 24 h-biofilm was treated with 20, 5 or 0 mg/ml NAC for 0 h or 4 h. After being collected and washed with PBS, cell pellets were sonicated to break the cell membrane and release intracellular NAC. The concentration of NAC was detected by an HPLC system equipped with a Phenomenex Luna C_18_ column (150 × 4.6 mm; 5 *μ*m, 100 Å) and an UV/visible detector set at 214 nm. (a) Retention time for NAC was at 1.660 min. A standard curve was made with NAC concentrations ranging from 0 to 1 mg/ml, and from this curve, peak areas are used to determine the concentration of NAC in the experiment. (b) After 20 mg/ml NAC treatment for 0 and 4 h, the concentrations of intracellular NAC were significantly higher than both control and 5 mg/ml NAC. Biological duplicates from each treatment were used for the quantitative data.

To determine whether NAC affects the bacteria by entering the cytosol and causing changes that potentially kill the bacteria, we used HPLC to determine whether NAC is found inside the bacteria after the treatments. NAC at 20 mg/ml kills the bacteria and at 5 mg/ml does not kill the bacteria. These concentrations were applied to fully develop chronic wound biofilm, and NAC inside the bacteria was analyzed at 0 and 4 h after its addition to the biofilm using HPLC (Figure 5). The peaks with NAC standard solution in PBS and PBS blank are shown in Figure 5(a). When the bacteria were treated with 20 mg/ml of NAC, we observed that a significant amount of NAC was found inside the bacteria both right after the treatment, indicating rapid penetration into the cells, and also 4 h later suggesting that it is retained inside the bacteria. Biofilm treated with 5 mg/ml NAC, however, showed no significant intracellular NAC in the bacteria when compared to the control (Figure 5(b)). These results suggest that in the presence of 20 mg/ml of NAC which has a pH < pKa, NAC can readily enter the cytosol, whereas NAC 5 mg/ml which has a pH above the pKa does not penetrate the bacteria cell membrane and is not found inside the cytosol.

**Figure 6:**
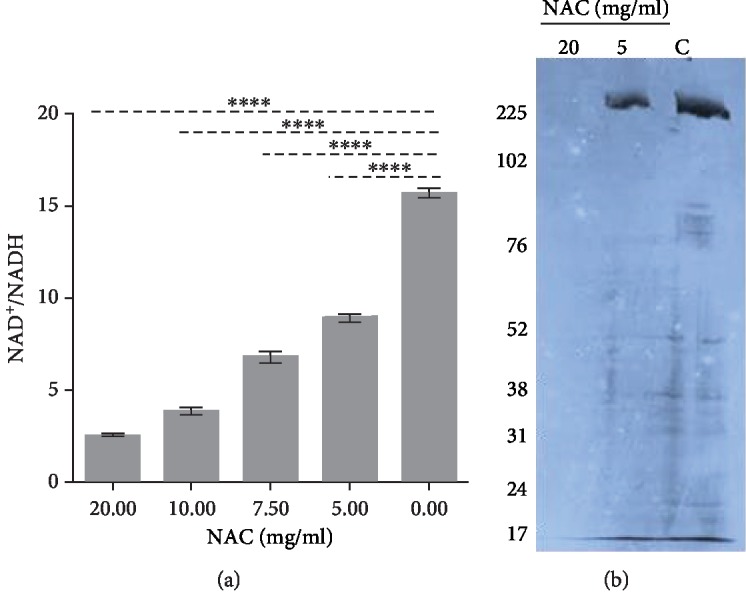
The effects of NAC on bacterial oxidative stress and protein synthesis. (a) NAD^+^/NADH ratio for the 24 h biofilm treated with NAC. The ratio decreases with the increase of NAC concentration indicating that NAC treatment causes an increase in oxidative stress in the cytosol of the bacteria. (b) ^35^S labeling of proteins shows that NAC at 20 mg/ml inhibits protein synthesis whereas at 5 mg/ml does not.

We also determined whether NAC affects oxidative stress inside the bacterial cells. NAD^+^ is an essential cofactor which is required for redox balance and energy metabolism [31]. The NAD^+^/NADH ratio is a key indicator of the redox state in the cell and of cell metabolism. In order to determine whether NAC at pH below the pKa alters the reduction potential of the bacteria, the NAD^+^/NADH ratio was measured after NAC treatment (Figure 6(a)). In the presence of NAC, the ratio of NAD^+^/NADH decreased in a dose/pH-dependent manner. For the control group, the NAD^+^/NADH ratio is around 15.7; in the presence of NAC 5, 7.5, 10, and 20 mg/ml, the NAD^+^/NADH ratio declined by 43.3%, 56.8%, 75.4%, and 84.1%, respectively (*P* < 0.0001). We then examined the effects of these concentrations of NAC on protein synthesis by ^35^S labeling and found that 20 mg/ml NAC completely stopped protein synthesis whereas 5 mg/ml NAC have a very little effect (Figure 6(b)). These results indicate that NAC, at pH below the pKa, interferes with the cellular redox states of the bacteria in the biofilm altering metabolic activity potentially leading to cell death.

### 3.5. NAC Interferes with Components of the Biofilm EPS

**Figure 7:**
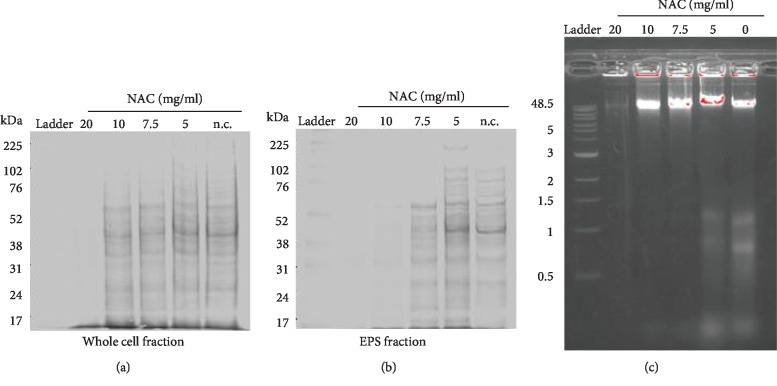
The effect of NAC on the protein and DNA of the EPS. 24 h chronic wound microbiome biofilm was treated with NAC at different concentrations for 24 h. The same amount of biofilm inoculum was extracted and analyzed on 12% SDS-PAGE gels. (a) Whole protein extract. (b) Soluble protein component (EPS fraction). In the presence of 20 mg/ml NAC, there were significantly less proteins both in the whole extract and in the soluble fraction. With 10 mg/ml NAC, the protein in the whole extract was not different from that of the control whereas the protein in the supernatant was significantly decreased. NAC treatment with less than of 7.5 mg/ml did not alter either the whole extract or the soluble proteins except for those above ~60 kDa. (c) DNA components in the biofilm matrix were analyzed using 1% agarose gels. A prominent band was seen at 45.8 kb with 5, 7.5, and 10 mg/ml NAC treatments. Treatment with 20 mg/ml of NAC reduced the amount of DNA present in that band, and no other DNA bands were seen. The pattern of DNA in the EPS with 5 mg/ml NAC treatment was similar to that of the control and showed not only the 45.8 kb band but also other smaller bands: <1.5 kb. These smaller bands were not seen with any of the other NAC treatments. We used biological triplicates for each treatment for SDS-PAGE gels.

In order to address whether NAC interferes with the integrity of the biofilm, we analyzed the composition of proteins and DNA in the biofilm. The same amount of inoculum from each treatment was collected and loaded on SDS-PAGE and agarose gels after treatment with different concentrations of NAC for 24 h (Figure 7**)**. In the presence of 20 mg/ml NAC, there were many protein bands present for the whole biofilm fraction, in particular, for large molecular weight proteins (Figure 7(a)). In the EPS fraction, there were fewer soluble proteins with 20 and 10 mg/ml NAC treatment, particularly in proteins above 52 kDa (Figure 7(b)). This effect was very clear for the 20 mg/ml NAC treatment. As for the biofilm DNA, in the absence of NAC, there were a number of strong bands below 2 kb and a strong band at 48.5 kb (Figure 7(c)). With 5 mg/ml NAC treatment, the pattern was very similar to that of the control. When 7.5 and 10 mg/ml NAC treatments were used, the smaller DNA bands (<1.5 kb) disappeared, whereas with 20 mg/ml NAC treatment not only the smaller bands disappeared but the 48.5 kb band was virtually gone. These results suggest that NAC can interfere with critical components of the biofilm leading to dismantling of the EPS.

**Figure 8:**
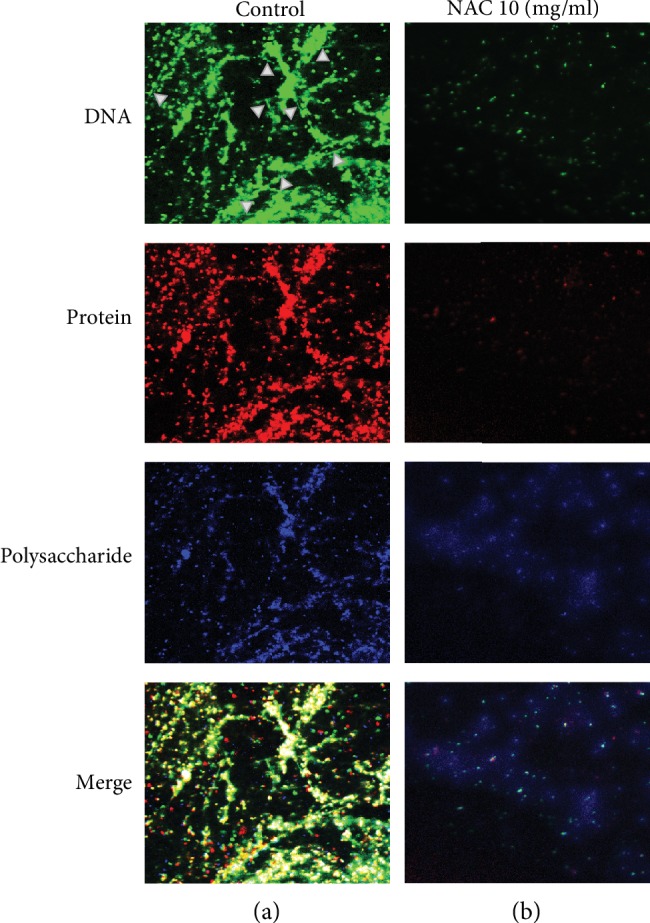
Fluorescence staining and confocal laser scanning microscopy analysis of the biofilm: DNA (green), proteins (red), and polysaccharides (blue) were visualized by staining with specific stains as described in the Methods and Materials section. Pictures in (a) show that without the application of NAC, extracellular DNA molecules appear as strings in the matrix (arrowheads). The proteins are primarily found either in the bacteria which were planktonic or in aggregates. The carbohydrates are mostly in association with the planktonic bacteria and the bacterial aggregates. Pictures in (b) show that treatment with 10 mg/ml NAC resulted in virtually no bacteria present and the EPS was found to be mostly gone. Biological duplicates for each treatment were used for the images.

To support the biochemical results, the architecture of the biofilm was further investigated using confocal laser scanning microscopy (Figures 8(a) and 8(b); control and NAC-treated, respectively). The contents of biofilm matrix DNA (green), proteins (red), and polysaccharides (blue) were visualized by staining with specific stains. Without the application of NAC, biofilm contained extracellular DNA molecules, which in the image appear as strings in the matrix (arrowheads). The proteins were primary found either in the bacteria which were planktonic or in aggregates. The carbohydrates were harder to define, but most of the staining was associated with the planktonic bacteria and the aggregates. Treatment with 10 mg/ml NAC resulted in virtually no bacteria present in the biofilm, and the EPS was found to be greatly decreased. These results taken together further support our hypothesis that NAC affects biofilm EPS matrix and bacteria survival.

### 3.6. Importance of Chemical Groups for NAC Function

**Figure 9:**
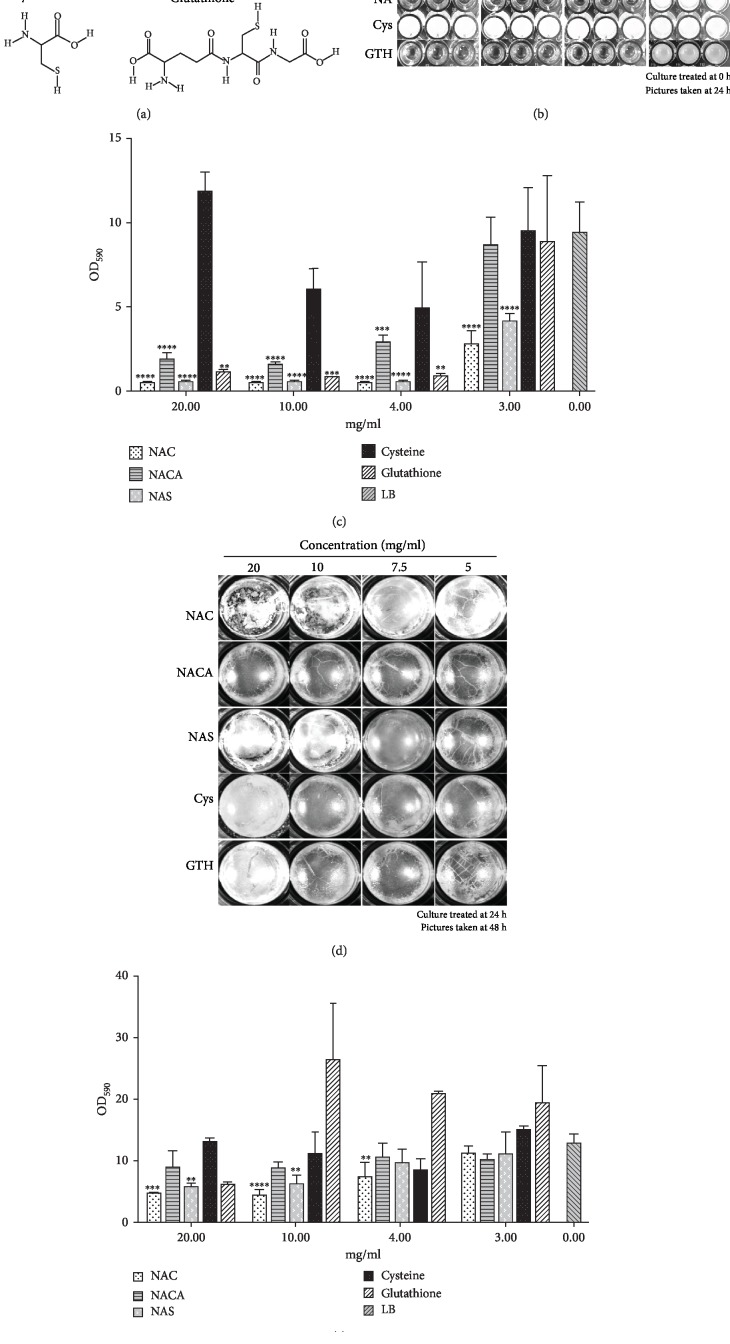
The effects of NAC and similar molecules in dismantling biofilm. (a) Structures of molecules similar to NAC used in this experiment: *N*-acetyl-cysteine amide (NACA), *N*-acetyl-serine (NAS), cysteine (Cys), and glutathione (GTH). (b) Chronic wound microbiome biofilm formation after the treatment at time 0 h with the different molecules with concentration ranging from 0 to 20 mg/ml. >4 mg/ml of NAC, NAS, and GTH interfered with chronic wound microbiome cell growth. However, the same amount of NACA and cysteine did not affect cell growth. (c) Quantification of the biofilm formation from (b) using crystal violet (CV) staining. Compared to LB media as the control, ≥4 mg/ml NAC, NAS, and GTH were able to stop the formation of biofilm. ≥4 mg/ml NACA did not affect cell growth in (b), yet significantly reduced biofilm. The application of 3-20 mg/ml Cys to chronic wound microbiome culture did not increase or reduce biofilm formation. (d) 24 h chronic wound microbiome biofilm was treated with various molecules and the effects recorded at 48 h. Biofilm was visually dismantled when treated with ≥10 mg/ml NAC and 20 mg/ml NAS. NACA, cysteine, and GTH did not seem to affect the biofilm. (e) Quantification of existing biofilm formation from (d) using CV staining. ≥7.5 mg/ml NAC and ≥10 mg/ml NAS can significantly decrease existing biofilm. Technical triplicates for each treatment for both cultured biofilm and quantitative data.

In order to determine whether the functional groups of NAC are crucial to its biofilm-dismantling functions, we tested molecules with analogous structure, including *N*-acetyl cysteine amide (NACA), *N*-acetyl serine (NAS), cysteine (Cys), and glutathione (GTH) (Figure 9(a)). NACA has an amino group that neutralizes the carboxyl group; NAS contains a hydroxyl group in place of the thiol group; Cys lacks the acetyl group; GTH is a more complex molecule than NAC, but it has a similar antioxidant effect as NAC. When ≥4 mg/ml of NAC, NAS, and GTH were applied at the beginning of the culture and observed 24 h later, there was no bacterial growth (Figure 9(b)). Similarly, there was virtually no significant crystal violet staining confirming the absence of biofilm attached to the well (Figure 9(c)). However, bacteria grew when the same concentrations of NACA and cysteine were applied (Figure 9(b)), but the development of biofilm was much less strong in the presence of ≥4 mg/ml of NACA than in the presence of Cys (Figure 9(c)).

When we tested the effects of these molecules on 24 h chronic wound biofilm and observed them 48 h later (Figures 9(d) and 9(e)), we observed that ≥10 mg/ml of NAC and NAS were able to disrupt surface biofilm integrity, and CV staining showed that ≥7.5 mg/ml of NAC decreased the amount of existing biofilm. Surface biofilm treated with ≥10 mg/ml NAS visibly became fragile, and CV staining showed similar results. NACA and Cys treatments did not disrupt biofilm visually or by CV staining. 20 mg/ml GTH reduced the existing biofilm by ~55%, but not with the lower concentrations (Figures 9(d) and 9(e)). These results suggest that the acetyl and carboxyl groups play important roles in NAC biofilm dismantling ability.

## 4. Discussion

NAC has been used extensively to treat excess mucus formation; in particular, it is very effective as a lung mucolytic in cystic fibrosis patients [32]. Because *P. aeruginosa* is critical in cystic fibrosis and also a very difficult bacteria to eradicate in the biofilms of human chronic wounds, we chose to study biofilm derived from chronic wound microbiome that contained primarily *P. aeruginosa*. Using biofilm from chronic wounds in humans is not justifiable at this time, because we need to first demonstrate that our *in vitro* system is reliable and reproducible. Indeed, our next step will be a proof-of-concept study with microbiome taken directly from human chronic diabetic foot ulcers.

We have recently shown that the application of NAC to chronic wounds in a diabetic mouse model we developed, in conjunction with systemic application of *α*-tocopherol, results in the disappearance of the bacteria from the biofilm and leads to the loss of the EPS. This in turn results in the healing of the wounds. This is the reason why we decided to use the microbiomes from the chronic wounds in this model to establish the biofilms *in vitro*. When applied to the biofilm *in vitro*, NAC killed bacteria, decreased biofilm formation, and dismantled existing biofilm. In the presence of NAC at a pH below the pKa, both chronic wound bacteria and the EPS in the biofilm were affected—bacteria did not survive, and the EPS began to fall apart and disperse. We also determined that the DNA and protein content of EPS were significantly decreased after treatment with NAC at pH below the pKa. Moreover, under these conditions, we found that NAC penetrates the bacterial membrane, increases the oxidative stress, and inhibits protein synthesis. When treating with molecules that share the basic structure of NAC but differ in the acetyl, thiol, and hydroxyl groups, we found that lack of the acetyl group, for example, in cysteine, prevented NAC from inhibiting bacterial cell growth and dismantling biofilm. Replacing the hydroxyl group in the carboxyl group with an amino group, as in NACA, resulted in similar effects. However, the exchange of the thiol group for a hydroxyl group did not interfere either with inhibition of bacterial cell growth or with biofilm dismantling. These latter results show that both the acetyl and the carboxylic groups in NAC are critical for NAC function. These results provide insights on how NAC interferes with biofilm structure and how it kills bacteria embedded in the EPS.

Weak organic acids have been used for antibacterial property for decades [33]. To quote, “An acidic environment created by the use of acid, such as acetic acid, boric acid, ascorbic acid, alginic acid, and hyaluronic acid, help in wound healing by controlling wound infection…” [34]. The treatment of the chronic wound with 1% (pH 2.75) to 5% acetic acid (pH 2.4) has been applied to ulcers and burn wounds and showed to be beneficial in eradicating infection and promoting wound healing [17, 35]. Also, 3% citric acid was used locally to treat a variety of chronic wounds such as diabetic foot infections and burn wound infections [36–38]. NAC is a weak acid and therefore negatively charged at physiological pH which makes it very difficult to penetrate into the biofilm and the bacteria. However, when NAC is at high concentrations, its pH is below the pKa rendering it able to penetrate the negatively charged biofilm and bacterial membrane. Thus, the pH is a critical factor for NAC penetration of the biofilm. 10 and 20 mg/ml NAC have pH below the pKa. These concentrations can kill the bacteria and dismantle the biofilm, and when compared to weak acid (acetic acid) and strong acid (HCL), NAC was more effective in dismantling biofilm at the same pH suggesting addition functions. This has also been confirmed in a laboratory-developed *P. aeruginosa* strain overproducing alginate—pH alters the size of the microcolonies and interferes with NAC bactericidal ability [39]. These results suggest that NAC interferes with the structure of extracellular matrix and then harms the cells within the biofilm.

Bacterial biofilm EPS contains proteins, nucleic acids, polysaccharides, and lipids, and its integrity greatly relies on close interactions among these components. It is known that for *P. aeruginosa*, the crosslinks of extracellular CdrA protein with polysaccharide Psl [40], the ionic interactions between polysaccharide Pel and eDNA [41], and the binding of eDNA released from cells to phenazines such as pyocyanin [42, 43] are the major players for its biofilm architecture. We are currently pursuing some of these lines of inquiry to determine if NAC interferes with any of these proteins.

In humans, NAC has been used as a mucolytic agent which depends on its ability to break down disulfide bridges in the glycoproteins and mucin of the mucus reducing its viscosity [44–46]. Disulfide bonds are also important for the stability of many extracellular and cytoplasmic proteins. In prokaryotes, disulfide bonds also play an important role in the stability of the extracellular substances. For example, *P. aeruginosa* secretes an array of proteins, many of which contain disulfide bonds, such as FapC, an amyloid protein abundant in *P. aeruginosa* EPS, which requires two highly conserved cysteines to form disulfide linkages between two monomers to form mature fibrils [47]. A 29 kDa extracellular lipase secreted by *P. aeruginosa* contains two cysteine residues to maintain its active conformation [48]. The elastase of *P. aeruginosa* requires disulfide bonds for the full proteolytic activity of the enzyme and structural stability [49]. Moreover, Gram-negative oral pathogens *Porphyromonas gingivalis* and *Tannerella forsythia* use disulfide bonds to stabilize their outer membrane porin proteins [50]. For *Actinomyces oris*, disulfide bond formation is needed for pilus assembly, coaggregation, and biofilm formation. Because of the importance of disulfide bonds in the stability of the EPS in general, but specifically in the *P. aeruginosa* EPS, we will be pursuing studies on how NAC interferes with disulfide bonds present within the EPS.

Oxidative state plays an important role in cell integrity and function, including bacteria [51, 52]. We show here that the NAC application to *P. aeruginosa* biofilm in our *in vitro* wound biofilm system leads to a reduced ratio of NAD^+^/NADH inside the bacteria, indicating high levels of oxidative stress [53]. After 24 h of treatment with NAC, the NAD^+^/NADH ratio significantly declines as the NAC concentration increases, which can potentially be due to the buildup of NADH or reduced levels of NAD^+^ leading to the increase of the intracellular oxidative status. During wound healing, low concentration of reactive oxygen species (ROS) is critical for proper healing. However, chronic wounds contain excessive ROS, which causes damage to the wound tissue [6]. Our results suggest that *in vivo* NAC significantly accelerates the healing process because it helps clear the wound biofilm and provides antioxidant properties to the cells in the wound tissue helping them survive and function [24].

Finally, we are currently undertaking the studies to examine the effects of NAC and its mechanism of action on biofilm-forming bacteria such as *Enterobacteriaceae* and *Staphylococcus*. These studies are designed to determine whether NAC affects other biofilm-forming bacteria in the same manner as it affects *P. aeruginosa*. In the end, when we understand better how NAC affects the various biofilm forming bacteria individually, we will be able to create complex biofilms *in vitro* to determine how to best use NAC to kill bacteria and dismantle wound biofilm and how to use it in combination with other drugs using high-throughput screening.

## 5. Summary

Chronic wounds cause a significant burden to individuals and the society. Using an *in vitro* biofilm system we developed and microbiome taken from chronic wounds, we show here that NAC at pH < pKa significantly improves the healing of chronic wound-containing biofilm by killing the bacteria and dismantling the EPS. We found that NAC penetrates the bacterial cell membrane, causes an increase in oxidative stress, and halts protein synthesis and that the acetyl and carboxylic groups of NAC play an important role in the effects of NAC on biofilm. Furthermore, NAC interferes with the proteins and DNA in the EPS leading to the dismantling of the biofilm. Using this system, we can perform a proof-of-concept study with biofilm taken directly from human chronic wounds and then develop the system for clinical and personalized medicine. Our findings can provide insights into the development of new therapeutics for the elimination of wound microbiome.

## Data Availability

Access to the data will be made without restrictions after the data is published.

## Abbreviation

Cys: Cysteine
EPS: Extracellular polymeric substance
GTH: Glutathione
LB: Luria broth
NAC: *N*-Acetyl-cysteine
NACA: *N*-Acetyl cysteine amide
NAS: *N*-Acetyl serine
ROS: Reactive oxygen species.

## Supplementary Materials

Supplementary Materials Effects of NAC on biofilm-free bacterial growth. To investigate the effect of NAC on biofilm-free bacterial growth, bacteria from the microbiome of chronic wounds were cultured and diluted so that individual colonies would form after plating on LB agar plates for 7 h inoculation (Supplementary Figure 1A, top panel). 5-20 mg/ml NAC were applied to the center of the colony patches. Colonies in the middle of the patch treated with 20 mg/ml remained to have the same size or became smaller at 10 h and 24 h, respectively (Supplementary Figure 1A, bottom panel). However, the colonies at the periphery of the circle where NAC was not added continued to grow in all conditions. We then collected bacteria from the center and the periphery of the cultures at 24 h of treatment with 20 mg/ml NAC and subcultured them in LB to determine if the bacteria would grow. There was no further growth of the colonies in the middle, which indicates that the bacteria were killed after NAC treatment (Supplementary Figure 1B). However, those present on the edge of the circle grew well. To determine whether a larger volume of NAC solution could kill the bacteria entirely, we applied more NAC solution in culture rings placed over the patch cultures on the LB agar at 7 h after the start of the culture (Supplementary Figure 1C). 20 mg/ml NAC were able to stop bacteria growth up to 48 h whereas 10 mg/ml NAC inhibited cell growth up to 24 h. 7.5 and 5 mg/ml NAC did not effectively prevent cell growth. These results suggest that NAC can inhibit bacterial cell growth independently of the presence of biofilm. Supplementary Figure 1: the bactericidal ability of NAC on chronic wound bacterial cells in the absence of biofilm. (A) 5 *μ*l of diluted chronic wound bacteria inoculum was plated and cultured for 7 h when individual colonies became visible. NAC at concentrations ranging from 5 to 20 mg/ml was applied by adding 5 *μ*l of the NAC solution on the top of the colonies in the center of the patch. Colony growth was recorded at 10 and 24 h; treatment with 20 mg/ml resulted in the disappearance of the colonies in the middle of the patch but not in the edges. (B) Technical duplicates for cell viabilities of the middle/side of the colony patch. The middle and edge of 24 h cell patches treated with 20 mg/ml of NAC were subcultured in fresh LB. 20 mg/ml of NAC prevented further cell growth in the middle (left two tubes); 10 and 5 mg/ml of NAC were not able to stop the growth of the colonies (right two tubes). (C) Culture rings were placed on top of the patches to allow for the application of a larger volume of NAC solution. 200 *μ*l 20 mg/ml NAC prevented further bacteria growth up to 48 h. 10 mg/ml of NAC inhibited cell growth up to 24 h; however, it failed to inhibit colony growth at the edge of the ring at 48 h. 7.5 and 5 mg/ml did not inhibit chronic wound bacteria growth. Biological triplicates for each treatment for cultured biofilm.
