# Supplementary material for: N-Acetyl-cysteine and Mechanisms Involved in Resolution of Chronic Wound Biofilm
Source: J Diabetes Res. 2020 Jan 27;2020:9589507. doi: 10.1155/2020/9589507 (PMC7007959; doi:10.1155/2020/9589507)
Supplement: Supplementary Materials — Effects of NAC on biofilm-free bacterial growth. To investigate the effect of NAC on biofilm-free bacterial growth, bacteria from the microbiome of chronic wounds were cultured and diluted so that individual colonies would form after plating on LB agar plates for 7 h inoculation (Supplementary Figure 1A, top panel). 5-20 mg/ml NAC were applied to the center of the colony patches. Colonies in the middle of the patch treated with 20 mg/ml remained to have the same size or became smaller at 10 h and 24 h, respectively (Supplementary Figure 1A, bottom panel). However, the colonies at the periphery of the circle where NAC was not added continued to grow in all conditions. We then collected bacteria from the center and the periphery of the cultures at 24 h of treatment with 20 mg/ml NAC and subcultured them in LB to determine if the bacteria would grow. There was no further growth of the colonies in the middle, which indicates that the bacteria were killed after NAC treatment (Supplementary Figure 1B). However, those present on the edge of the circle grew well. To determine whether a larger volume of NAC solution could kill the bacteria entirely, we applied more NAC solution in culture rings placed over the patch cultures on the LB agar at 7 h after the start of the culture (Supplementary Figure 1C). 20 mg/ml NAC were able to stop bacteria growth up to 48 h whereas 10 mg/ml NAC inhibited cell growth up to 24 h. 7.5 and 5 mg/ml NAC did not effectively prevent cell growth. These results suggest that NAC can inhibit bacterial cell growth independently of the presence of biofilm. Supplementary Figure 1: the bactericidal ability of NAC on chronic wound bacterial cells in the absence of biofilm. (A) 5 μl of diluted chronic wound bacteria inoculum was plated and cultured for 7 h when individual colonies became visible. NAC at concentrations ranging from 5 to 20 mg/ml was applied by adding 5 μl of the NAC solution on the top of the colonies in the center of the patch. Colony grow [file 9589507.f1.pdf]

Effects of NAC on biofilm-free bacterial growth

To investigate the effect of NAC on biofilm-free bacterial growth, bacteria from the microbiome of chronic wounds were cultured and diluted so that individual colonies would form after plating on LB agar plates for 7h (**Supplementary Fig.1A, top panel**). 5-20mg/ml NAC were applied to the center of the colony patch. Colonies in the middle of the patch treated with 20mg/ml remained the same size or became smaller at 10h and 24h, respectively (**Supplementary Fig.1A, bottom panel**). However, the colonies at the periphery of the circle where NAC was not added continued to grow in all conditions. We then collected bacteria from the center and the periphery of the cultures at 24h of treatment with 20mg/ml NAC, and sub-cultured them in LB to determine if the bacteria would grow. There was no further growth of the colonies in the middle, which indicates that the bacteria were killed after NAC treatment (**Supplementary Fig. 1B**). However, those present on the edge of the circle grew well. To determine whether a larger volume of NAC solution could kill the bacteria entirely, we applied more NAC solution in culture rings placed over the patch cultures on the LB agar at 7h after the start of the culture (**Supplementary Fig. 1C**). 20mg/ml NAC were able to stop bacteria growth up to 48h whereas 10mg/ml NAC inhibited cell growth up to 24h. 7.5 and 5mg/ml NAC did not effectively prevent cell growth. These results suggest that NAC can inhibit bacterial cell growth independently of the presence of biofilm.

Supplementary Figure 1. The bactericidal ability of NAC on chronic wound bacterial cells in the absence of biofilm. (**A**) 5µl of diluted chronic wound bacteria inoculum were plated and cultured for 7h when individual colonies became visible. NAC at concentrations ranging from 5 – 20 mg/ml were applied by adding 5µl of the NAC solution on the top of the colonies in the center of the patch. Colony growth was recorded at 10 and 24h; treatment with 20mg/ml resulted

in disappearance of the colonies in the middle of the patch but not in the edges. (**B**) Technical duplicates for cell viabilities of the middle/side of the colony patch. The middle and edge of 24h-cell patches treated with 20mg/ml of NAC were sub-cultured in fresh LB. 20 mg/ml of NAC prevented further cell growth in the middle (left two tubes); 10 and 5 mg/ml of NAC were not able to stop the growth of the colonies (right two tubes). (**C**) Culture rings were placed on top of the patches to allow for the application of a larger volume of NAC solution. 200 µl 20 mg/ml NAC prevented further bacteria growth up to 48 h. 10 mg/ml of NAC inhibited cell growth up to 24 h; however, it failed to inhibit colonies growth at the edge of the ring at 48 h. 7.5 and 5 mg/ml did not inhibit chronic wound bacteria growth. Biological triplicates for each treatment for cultured biofilm.

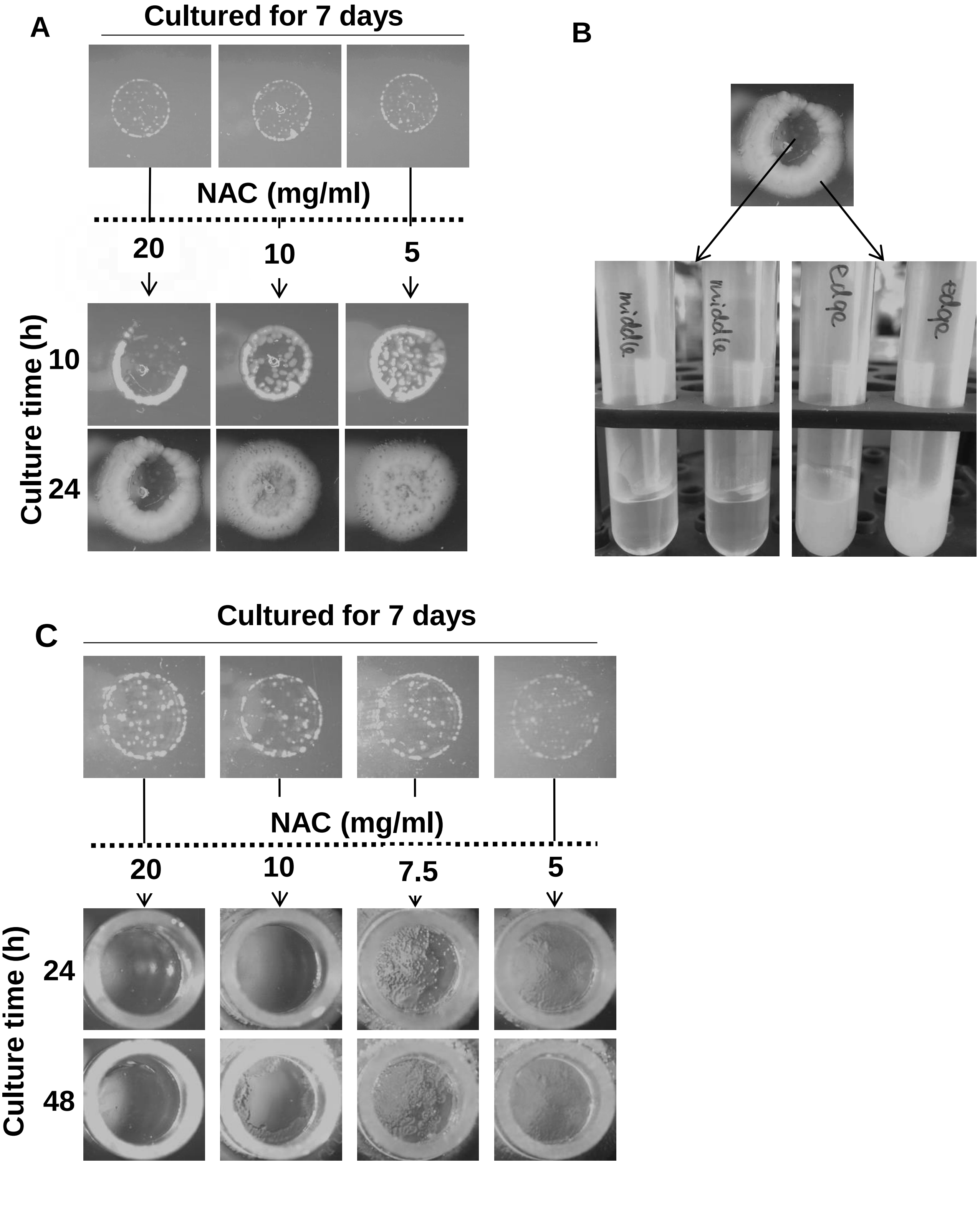

Supplementary Figure 1
